# Atypical Presentation of a Malignant Tumor of the Appendix: A Case Report

**DOI:** 10.7759/cureus.108201

**Published:** 2026-05-03

**Authors:** Maria H Garcia Ramírez, Jose M Zepeda Torres, Luis O Suárez Carreón, Laura B Alegría López, Estefanía Íñiguez Muñoz, Rodrigo Hernández Ramírez, José de J Murrieta Vallejo, Víctor M Ulloa Ulloa, Daniel A Ángel Montoya, Juan G García González

**Affiliations:** 1 Surgery, Specialty Hospital, Western Medical Center, Mexican Social Security Institute (IMSS), Guadalajara, MEX; 2 Bariatric Surgery, Specialty Hospital, Western Medical Center, Mexican Social Security Institute (IMSS), Guadalajara, MEX; 3 Coloproctology, Specialty Hospital, Western Medical Center, Mexican Social Security Institute (IMSS), Guadalajara, MEX; 4 General Surgery, Specialty Hospital, Western Medical Center, Mexican Social Security Institute (IMSS), Guadalajara, MEX; 5 Oncologic Surgery, Mexican Social Security Institute (IMSS), Morelia, MEX

**Keywords:** appendiceal adenocarcinoma, metastasis, mucinous tumor, rare tumour, surgical treatment

## Abstract

A 56-year-old man presented with weight loss, abdominal pain, and altered bowel habits. Computed tomography revealed an appendiceal mass with hepatic lesions and regional lymphadenopathy, suggestive of advanced appendiceal malignancy. Surgical exploration identified a mucin-producing appendiceal tumor with metastatic involvement of the mesentery, liver, and lymph nodes. Histopathological examination confirmed an invasive, moderately differentiated mucinous adenocarcinoma of the appendix, without lymphovascular or perineural invasion. The mesenteric lesion showed malignant neoplastic cells consistent with metastatic appendiceal adenocarcinoma, infiltrating the surrounding tissue. The patient underwent surgical resection followed by systemic chemotherapy. During follow-up, clinical evolution was consistent with advanced metastatic disease requiring ongoing multidisciplinary management. This case highlights the diagnostic and therapeutic challenges of advanced appendiceal mucinous adenocarcinoma and emphasizes the importance of complete pathological characterization and coordinated oncologic care.

## Introduction

Appendiceal adenocarcinoma is a rare malignant epithelial neoplasm arising from the mucosal lining of the vermiform appendix, accounting for less than 1% of all gastrointestinal tumors [1-3]. The World Health Organization currently classifies appendiceal adenocarcinomas into mucinous and nonmucinous (colonic-type) subtypes [1]. Mucinous appendiceal adenocarcinomas are characterized histologically by pools of extracellular mucin containing neoplastic epithelial cells, which may display low- or high-grade cytology and areas of infiltrative invasion [1]. Prognosis in appendiceal adenocarcinoma varies according to tumor grade, stage, histologic subtype, and extent of metastatic disease, with poorer outcomes generally observed in advanced-stage or high-grade tumors [1,4]. This case is noteworthy because it illustrates an advanced presentation of appendiceal mucinous adenocarcinoma with synchronous metastatic disease, highlighting the diagnostic difficulty created by nonspecific symptoms and the importance of correlating radiologic, surgical, and histopathologic findings to guide multidisciplinary treatment.

## Case presentation

A 56-year-old male with no significant personal medical history presented with a three-month history of constipation, abdominal pain, hyporexia, and unintentional weight loss of 20 kg. Colonoscopy showed no visible abnormality at the appendiceal orifice and no obvious intraluminal colorectal primary lesion, supporting its role in excluding an alternative colonic origin and complementing the radiologic suspicion of appendiceal malignancy. A contrasted abdominopelvic CT scan showed an appendiceal tumor, along with evidence of lymph node and liver metastases. These findings contributed to obstructive uropathy of the right kidney.

**Figure 1:**
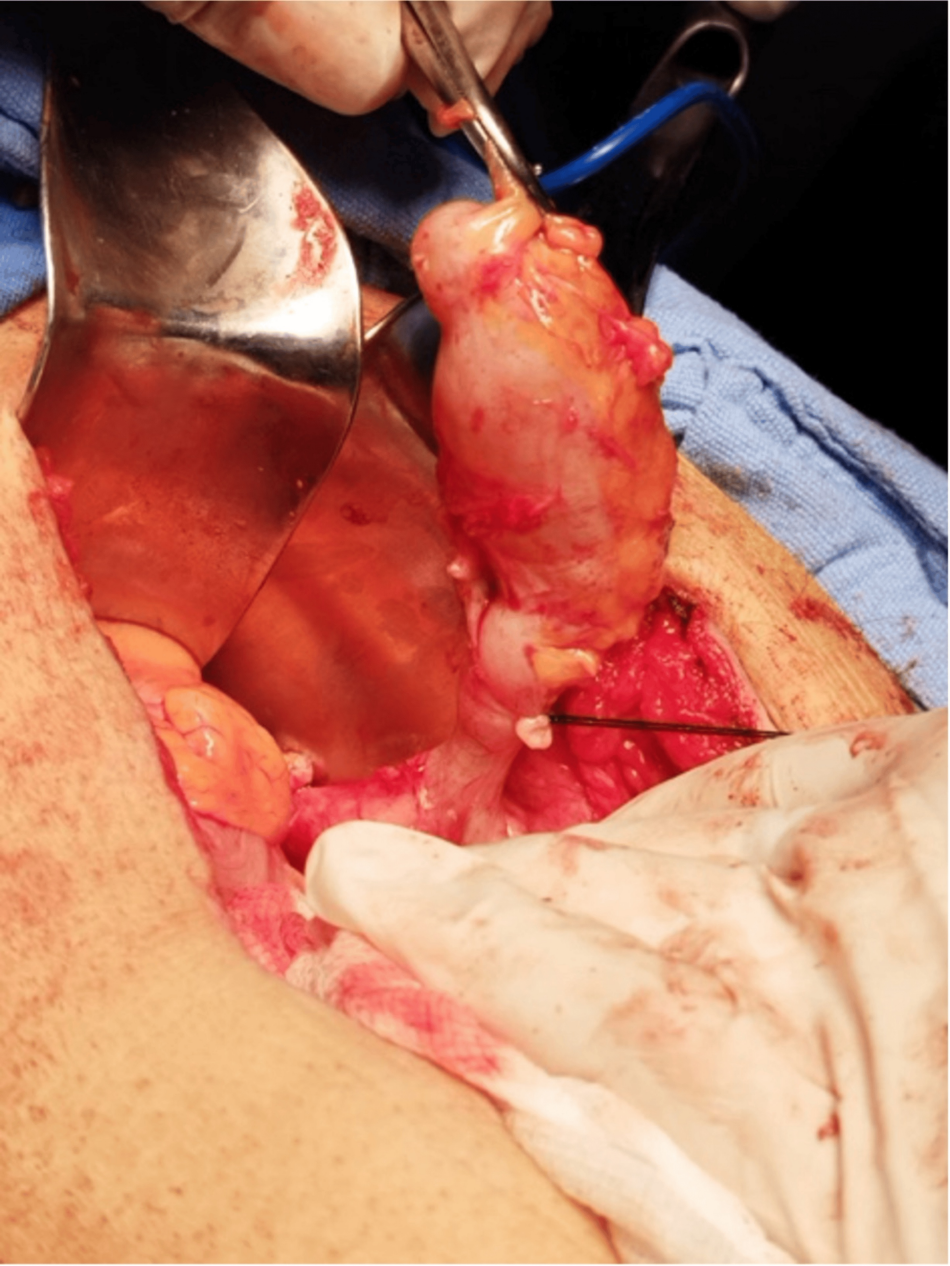
Appendicular tumor.

**Figure 2:**
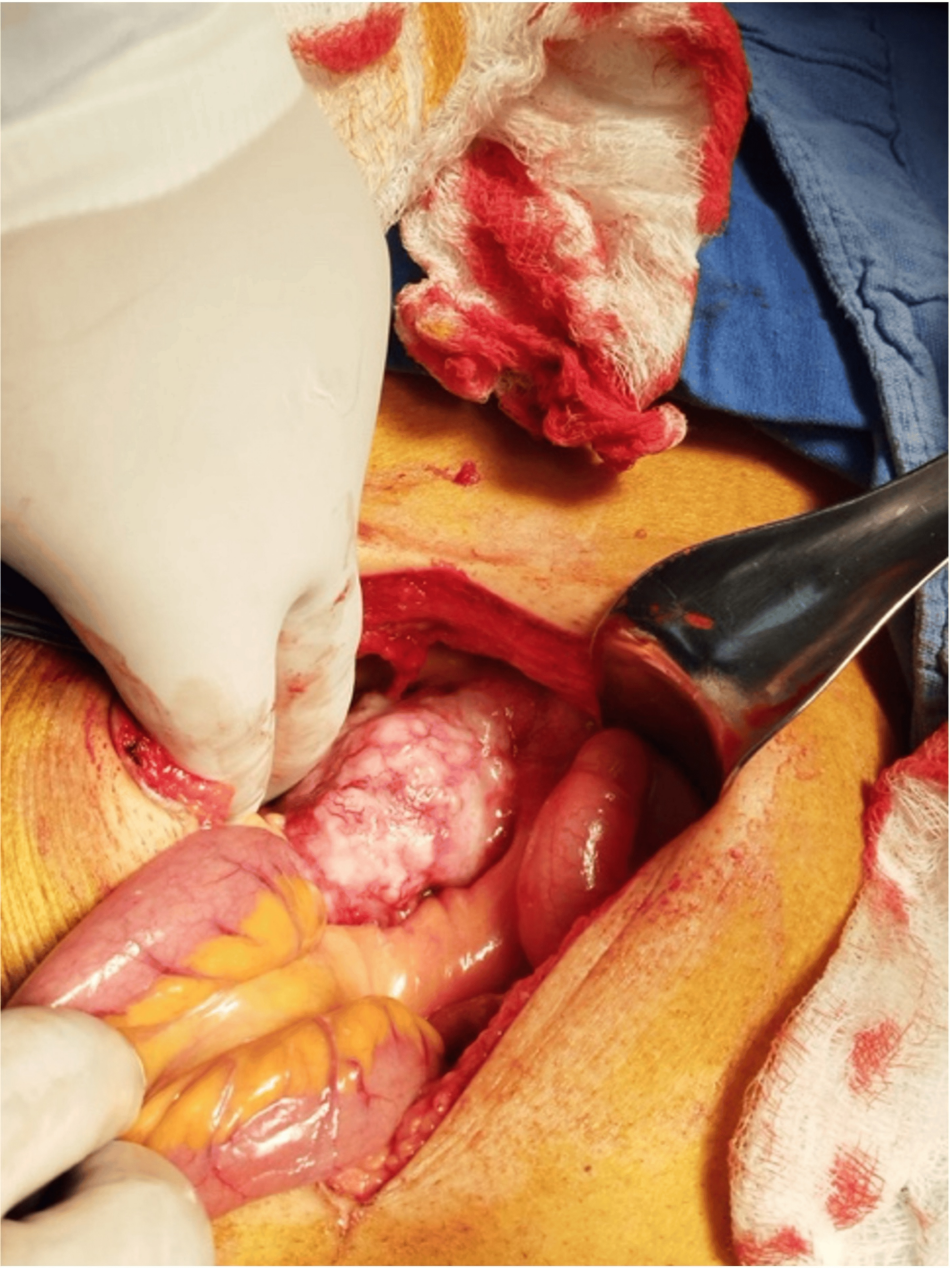
Stony tumor with cerebroid appearance in the mesenteric root.

**Figure 3:**
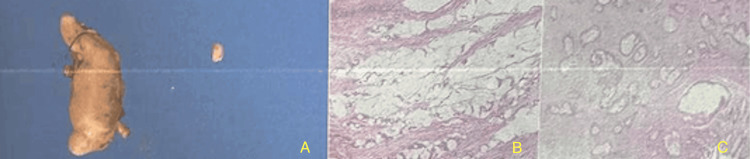
(A) Product of appendectomy; hematoxylin-eosin-saffron (HES) histological sections of (B) the appendiceal tumor and (C) the mesenteric tumor.

An exploratory laparotomy was conducted, which revealed a mucinous appendiceal tumor, considered the primary lesion. A separate firm lesion was identified at the mesenteric root and was interpreted as metastatic involvement, later confirmed histologically as malignant neoplastic cells consistent with appendiceal adenocarcinoma (Figures 1, 2). An appendectomy was performed, and a biopsy of the suspected metastatic lesion at the mesenteric root was taken. Intraoperative examination confirmed malignancy (Figure 3). Based on these findings, a right hemicolectomy was performed.

The patient was subsequently referred to Medical Oncology and diagnosed with appendiceal adenocarcinoma, clinical stage IV, due to the presence of liver and retroperitoneal lymph node metastases.

Histopathological examination of the appendix revealed an invasive mucinous adenocarcinoma, moderately differentiated, corresponding to grade 2 disease according to the current three-tier grading system for appendiceal adenocarcinomas. No lymphovascular invasion or perineural invasion was identified. The mesenteric lesion consisted of malignant neoplastic cells morphologically consistent with metastatic appendiceal adenocarcinoma, demonstrating infiltration of the surrounding tissue. The mitotic index was not specified in the original pathology report; however, tumor grade was reported as moderately differentiated (G2). No signet-ring cell component was described.

## Discussion

Molecular profiling has revealed that appendiceal adenocarcinomas are biologically distinct from colorectal carcinomas, with a unique spectrum of mutations. The most frequently mutated genes include RAS, GNAS, and TP53, with the distribution of these mutations varying by tumor grade and histology [5,6]. For example, well- and moderately differentiated tumors are more likely to harbor RAS and GNAS mutations, while poorly differentiated tumors more commonly have TP53 mutations [6]. These molecular subtypes are associated with differences in clinical behavior, metastatic potential, and response to chemotherapy [5].

Clinically, appendiceal adenocarcinomas often present with nonspecific symptoms, such as those mimicking acute appendicitis, or are discovered incidentally during surgery or imaging for unrelated conditions [1-3]. Advanced cases may present with peritoneal dissemination, sometimes manifesting as pseudomyxoma peritonei, particularly in mucinous subtypes [1,2].

Management depends on histologic subtype and stage. For invasive adenocarcinomas, oncologic right hemicolectomy is generally recommended due to the risk of lymphatic and hematogenous spread [2,4]. In contrast, localized, in situ lesions may be adequately treated with appendectomy alone [3]. Systemic chemotherapy is considered for advanced-stage disease, although its benefit is extrapolated from colorectal cancer data, as prospective randomized trials specific to appendiceal neoplasms are lacking [2].

Goblet cell adenocarcinoma, a distinct entity with amphicrine differentiation, is now recognized as a separate diagnosis due to its unique morphologic and clinical features [7,8]. High-grade variants, such as adenocarcinoma ex-goblet cell carcinoid, are particularly aggressive and frequently associated with peritoneal dissemination [8].

Treatment of appendiceal adenocarcinoma is determined by histologic subtype, tumor stage, and presence of peritoneal or distant metastases. For localized, early-stage (T1) appendiceal adenocarcinoma, recent multicenter data indicate that simple appendectomy may be sufficient, as right hemicolectomy does not confer a significant survival advantage in the absence of lymphovascular invasion or other high-risk features; lymph node metastasis in T1 disease is rare [9]. However, for more advanced local disease (T2 or higher), or when there is evidence of lymphovascular invasion, right hemicolectomy with oncologic lymphadenectomy is generally recommended to ensure adequate resection and staging, as these tumors can metastasize via lymphatics and bloodstream [10].

For mucinous and non-mucinous appendiceal adenocarcinomas with peritoneal dissemination, cytoreductive surgery (CRS) combined with hyperthermic intraperitoneal chemotherapy (HIPEC) is the standard of care, as this approach is associated with improved survival and disease control, particularly in well-differentiated and mucinous histologies [3-6]. In cases of perforated appendiceal adenocarcinoma, especially when peritoneal metastases are not initially identified, prophylactic CRS and HIPEC may reduce recurrence rates and improve peritoneal disease-free survival [11].

Systemic chemotherapy is generally reserved for advanced (stage III/IV) or unresectable disease, or when there is nodal or distant metastasis. Regimens are typically extrapolated from colorectal cancer protocols, most commonly using fluoropyrimidine-based combinations (e.g., FOLFOX, FOLFIRI, with or without bevacizumab), although the evidence for efficacy is limited and response rates are modest, especially in low-grade mucinous tumors [10,12-14]. Recent retrospective data suggest that taxane-based chemotherapy may have activity in metastatic appendiceal adenocarcinoma, warranting further investigation [7]. Notably, low-grade mucinous tumors are often indolent and relatively chemoresistant, and systemic chemotherapy has not demonstrated clear benefit in this subgroup [12].

## Conclusions

Appendiceal adenocarcinoma is an exceedingly rare but clinically significant malignancy with distinct histologic and molecular characteristics that influence its behavior and management. Precise histological classification, coupled with an understanding of its molecular profile, is essential for tailoring appropriate treatment strategies. Early-stage disease may be managed effectively with appendectomy alone, whereas advanced or high-risk tumors generally require more aggressive approaches such as right hemicolectomy combined with CRS and HIPEC, particularly when peritoneal dissemination is present. Systemic chemotherapy plays a role in advanced cases, although its efficacy remains variable, especially in low-grade mucinous subtypes. Ongoing research into the molecular pathways and targeted therapies holds promise for improving outcomes in patients with this rare neoplasm. A multidisciplinary approach remains crucial to optimize prognosis and quality of life for affected individuals.
